# Regionalization and Shaping Factors for Microbiomes and Core Resistomes in Atmospheric Particulate Matters

**DOI:** 10.1128/msystems.00698-22

**Published:** 2022-09-26

**Authors:** Ziyun Li, Mei Li, Shuhui Tong, Meng Fang, Weiwei Li, Ling Li, Xiang Li, Hai Xu, Xiaomin Sun, Mingyu Wang

**Affiliations:** a State Key Laboratory of Microbial Technology, Shandong University, Qingdao, China; b Environment Research Institute, Shandong University, Qingdao, China; c Department of Environmental Science and Engineering, Fudan University, Shanghai, China; d Institute of Mass Spectrometer and Atmospheric Environment, Jinan University, Guangzhou, China; e Guangdong Provincial Engineering Research Center for On-line Source Apportionment System of Air Pollution, Guangzhou, China; Zhejiang University School of Medicine

**Keywords:** antimicrobial resistance, atmospheric particulate matter, bacterial pathogenesis, microbiome, resistome, shaping factors

## Abstract

Antimicrobial resistance (AMR) seriously threatens public health by reducing antibiotic effectiveness in curing bacterial infections. Atmospheric particulate matter (APM) is a common environmental hazard that affects human health by causing various diseases and disseminating bacterial pathogenesis, of which pathogenic bacteria and AMR are essential parts. The properties of APM microbiomes and resistomes, along with their shaping factors and mutual relationships, need further examination. To address this, we analyzed APMs collected from 13 cities within four clusters (North and South China, Inner Mongolia, and Tibet). Significant regionalization was found for both the microbiomes (*P* < 0.001) and core resistomes (*P* < 0.001) for APMs, with statistical analyses showing significant differences in different regions. Principal coordinate analysis (PCoA) and accompanying ANOSIM analyses showed that microbiomes and core resistomes followed the same regional subclustering hierarchy patterns. This finding, together with response analysis of APM microbiomes and core resistomes to environmental parameters that showed similar response patterns, as well as Procrustes analysis (M^2^ = 0.963, *P* < 0.05) between APM microbiomes and core resistomes, strongly suggested that APM microbiomes and core resistomes are correlated. Co-occurrence network analysis further revealed key taxa and antimicrobial resistance determinants in the interactions between APM microbiomes and core resistomes. Thus, it was concluded that APM microbiome and resistome compositions were highly regional, that environmental pollutants and APM levels impacted APM microbiomes and resistomes, and that microbiomes and resistomes in APMs are significantly correlated (*P* < 0.05).

**IMPORTANCE** Bacteria associated with atmospheric particulate matter (APMs) can transmit over long distances. A large portion of these bacteria can potentially threaten human health. The antimicrobial resistance (AMR) of pathogenic bacteria carried by APMs prevents curing from infections. Therefore, both the pathogenic bacteria in APMs and their AMR are receiving more attention. The literature suggests a knowledge gap that exists for bacterial AMR and bacterial pathogenesis in APMs, including their distribution patterns, mutual relationships, and factors influencing their compositions. This work aimed to bridge this knowledge gap by studying APM samples collected from 13 cities. The results demonstrated that both bacteria and antibiotic resistance determinants were highly regional and that their composition patterns were significantly correlated, and influenced by the same group of environmental factors. This study thus determined the relationship between the two important aspects of bacterial pathogenesis in APMs and represents significant progress in understanding bacterial pathogenesis in APMs.

## INTRODUCTION

Atmospheric particulate matter (APM), a complicated mixture of inorganic, organic, and biological components, adversely affects human health by causing respiratory diseases (1–3), otitis media (4, 5), cerebrovascular, cardiovascular (1, 6, 7), renal (8), and neurological diseases (9). Evidence has shown that exposure to APM with aerodynamic diameters smaller than 2.5 μm (PM_2.5_) caused 4.02 million deaths worldwide in 2015 (10). Lelieveld et al. (11) used a global atmospheric chemistry model to predict worldwide annual premature deaths of 3.3 million to outdoor pollutants dominated by PM_2.5_.

Numerous studies have shown the presence of complex microbiome compositions in APM (12–16). Essentially all types of microbes, including bacteria, fungi, viruses, and archaea, can be found in APM, of which a large fraction can potentially affect human health (17–19). It was shown that live APM-associated bacterial pathogens, such as Klebsiella, *Aeromonas*, and *Bacillus*, could lead to respiratory diseases (16, 20, 21). These may transfer over long distances with APM (13), severely exacerbating the risks posed by airborne bacterial infections.

Antibiotics, the most influential medicine in the history of humans, are the primary tools to treat or prevent bacterial infections. Their misuse and overuse have led to the emergence of antimicrobial resistance (AMR), which has been listed as a global emerging environmental issue by the United Nations Environment Program (22). AMR is a significant player in accelerating the failure of antibiotics (23, 24). Recent investigations provided strong evidence that APMs are vehicles for AMR (16, 21, 25, 26), potentially aggravating bacterial pathogenesis. In addition to the environmental microbiomes of soil (27, 28), surface water (29, 30), urban wastewater treatment plants (31, 32), hospitals (33), and seawater (34), antibiotic resistance determinants (ARDs) were also widespread in airborne microbiomes (35, 36). ARDs of most of the common and last-line antibiotics, including colistin and vancomycin, have been detected in the air (16). Therefore, airborne particles have been proposed to be a pathway for the transmission of AMR (16).

Today, investigations on pathogenic bacteria and AMR carried by APMs are generally limited to the isolation of pathogens, investigations on airborne microbiomes, antibiotic resistance gene (ARG) levels, and resistomes in APMs. However, detailed studies on the shaping factors of APM microbiomes and resistomes, together with their mutual relationships, are still needed to understand better how bacterial pathogenesis is transmitted with APMs. Aiming at this question, we performed an extensive quantification, regionalization analysis, correlation analysis, and determination of shaping factors for APM microbiomes and core resistomes sampled in Northern and Southern China, Inner Mongolia, and Tibet. The results of this work contribute to our understanding of APM-mediated bacterial pathogenesis.

## RESULTS

### APM sampling and microbiome/core resistome determination.

**FIG 1:**
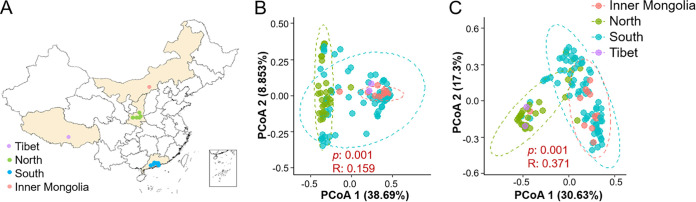
Sampling locations and regionalization of microbiome and core resistome compositions. (A) Sampling locations. (B) PCoA of microbiome compositions. (C) PCoA of core resistome compositions.

A total of 112 urban APM samples were collected in 13 Chinese cities, including five cities in Northern China (Baoji, Xi’an, Xianyang, Weinan, and Tongchuan of Shaanxi Province), six cities in Southern China (Guangzhou, Foshan, Shenzhen, Heshan, Dongguan of Guangdong Province, and Hong Kong Special Administration Region), and two outlying cities (Lhasa of Tibet Autonomous Region and Hohhot of Inner Mongolia Autonomous Region) (Fig. 1A and Table S1). Bacterial microbiomes were determined for each sample by high throughput 16S rRNA gene amplicon sequencing (Fig. S1A and Table S4). Core resistomes were determined using quantitative real-time PCR of 32 most common and important ARDs, including ARGs for quinolones, β-lactams, chloramphenicol, sulfonamides, tetracyclines, polymyxin, aminoglycosides, erythromycin, carbapenems, vancomycin, and three integrase-coding genes (Fig. S1B and Table S2 and S5). 16S rRNA gene levels were also quantified.

10.1128/msystems.00698-22.1 FIG S1 Microbial community structures and core resistome compositions in core atmospheric particulate matters. (A) microbial community structures. (B) Core resistomes compositions. BJ, Baoji; XA, Xi’an; XY, Xianyang; WN, Weinan; TC, Tongchuan; HK, Hong Kong Special Administrative Region; NMG, Inner Mongolia Autonomous Region; GZ, Guangzhou; FS, Foshan; SZ, Shenzhen; HS, Heshan; DG, Dongguan; XZ, Tibet. Download FIG S1, TIF file, 2.8 MB. Copyright © 2022 Li et al. 2022 Li et al. https://creativecommons.org/licenses/by/4.0/ This content is distributed under the terms of the Creative Commons Attribution 4.0 International license.

10.1128/msystems.00698-22.3 TABLE S1 Detailed information on APM samples. Download Table S1, XLSX file, 0.02 MB. Copyright © 2022 Li et al. 2022 Li et al. https://creativecommons.org/licenses/by/4.0/ This content is distributed under the terms of the Creative Commons Attribution 4.0 International license.

10.1128/msystems.00698-22.4 TABLE S2 qPCR primers used in this study. Download Table S2, DOCX file, 0.02 MB. Copyright © 2022 Li et al. 2022 Li et al. https://creativecommons.org/licenses/by/4.0/ This content is distributed under the terms of the Creative Commons Attribution 4.0 International license.

10.1128/msystems.00698-22.5 TABLE S3 Standard curves for qPCR reactions in this study. Download Table S3, DOCX file, 0.01 MB. Copyright © 2022 Li et al. 2022 Li et al. https://creativecommons.org/licenses/by/4.0/ This content is distributed under the terms of the Creative Commons Attribution 4.0 International license.

10.1128/msystems.00698-22.6 TABLE S4 Microbiome composition of each APM sample. Download Table S4, XLSX file, 0.2 MB. Copyright © 2022 Li et al. 2022 Li et al. https://creativecommons.org/licenses/by/4.0/ This content is distributed under the terms of the Creative Commons Attribution 4.0 International license.

10.1128/msystems.00698-22.7 TABLE S5 Normalized levels of ARDs in APM samples. Download Table S5, XLSX file, 0.1 MB. Copyright © 2022 Li et al. 2022 Li et al. https://creativecommons.org/licenses/by/4.0/ This content is distributed under the terms of the Creative Commons Attribution 4.0 International license.

### Geological regionalization of APM microbiomes and core resistomes.

To understand whether APM microbiomes and core resistomes were geologically regional, PCoA and analysis of similarities (ANOSIM) were performed. Significantly larger differences between groups were observed than within each group when microbiomes and core resistomes were grouped based on their geological locations (*P* < 0.001) (Fig. 1B and C and Fig. S1). These results strongly suggested that both APM microbiomes and core resistomes were regional. APMs from different places had different microbiomes and core resistomes, while APMs from the same place generally had similar microbiomes and core resistomes.

**FIG 2:**
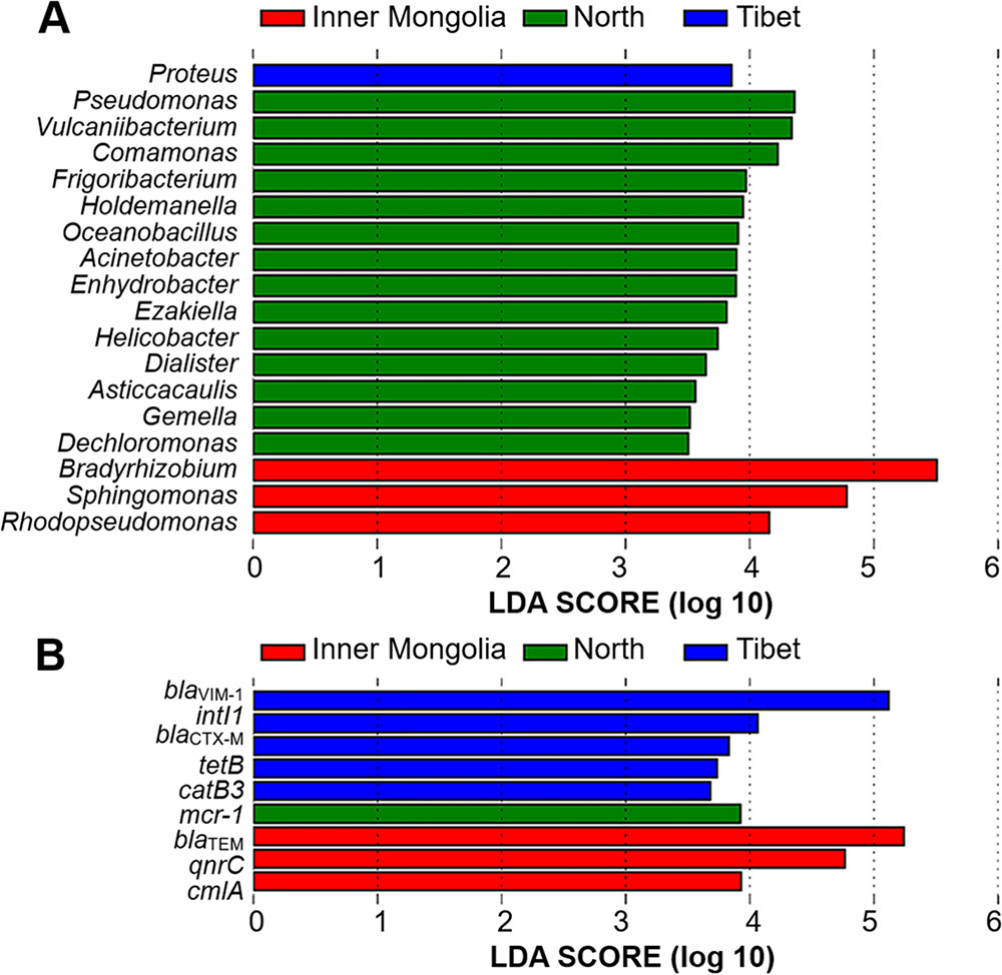
Microbial and ARG biomarkers of APM samples from different locations. (A) Microbial biomarkers at the genus level. (B) ARG biomarkers. Analysis was performed with LefSe analysis.

The enrichment of bacterial taxa and ARGs in different geological clusters analyzed with Linear discriminant analysis (LDA) effect size Linear discriminant analysis effect size (LEfSe) (Fig. 2) showed biomarkers for each geological group in APM microbiomes (Fig. 2A) and core resistomes (Fig. 2B). Notably, different opportunistic pathogens, including Proteus (Tibet), Acinetobacter, and Helicobacter (Northern China) were found to be enriched in different regions. The interesting enrichment of different ARGs for the same antibiotics in different regions further suggested the regional variations of resistomes in APMs. For β-lactams, resistomes in Southern China were characterized by the enrichment of *bla*_VIM-1_ and *bla*_CTX-M_, while resistomes in Inner Mongolia were characterized by the enrichment of *bla*_TEM_. For chloramphenicol, resistomes in Southern China were characterized by the enrichment of *catB3*, while resistomes in Inner Mongolia are characterized by the enrichment of *cmlA*.

**FIG 3:**
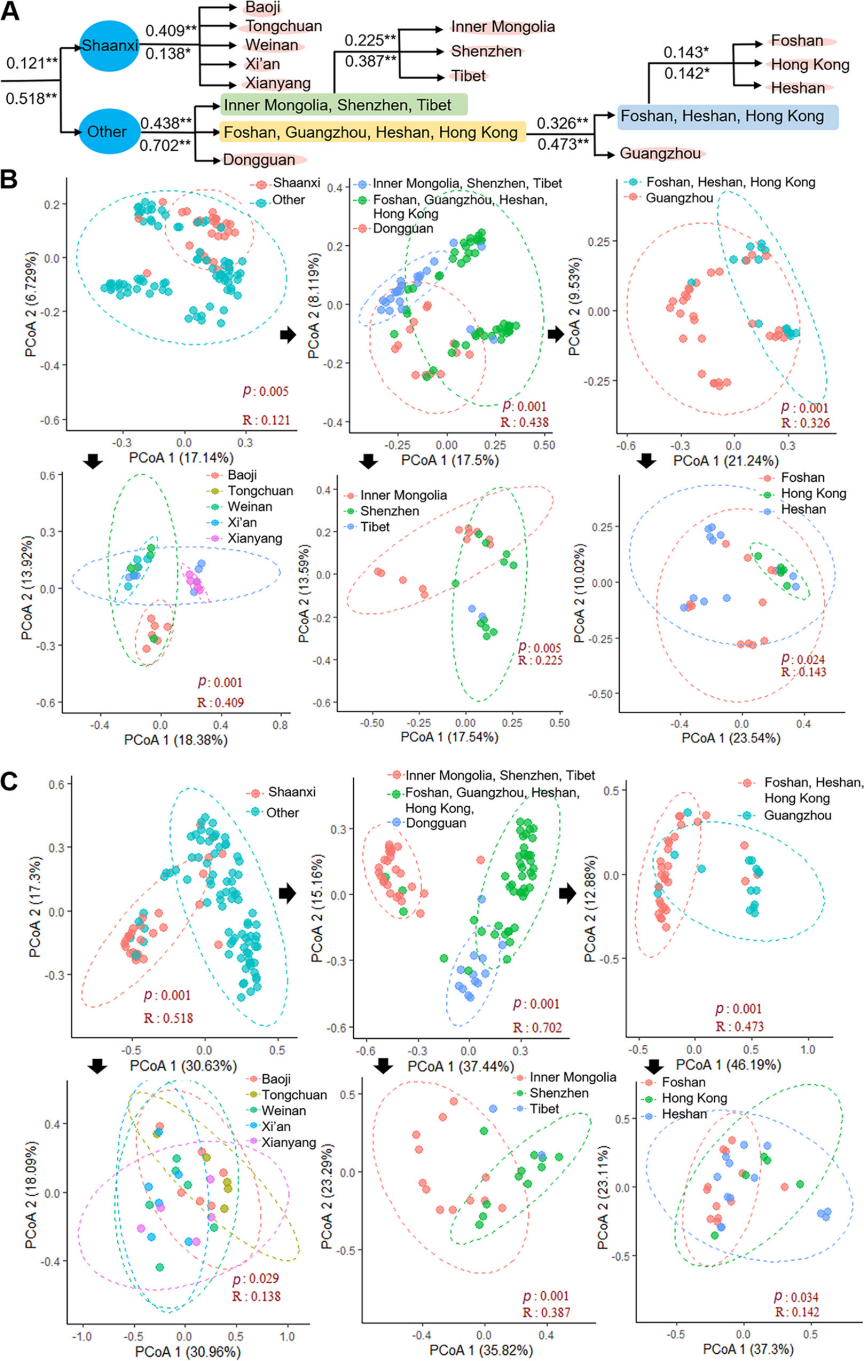
Regional subclustering of APM microbiomes and core resistomes. (A) regional subclustering of microbiomes and core resistomes. The numbers above and below the horizontal line indicate the R values for microbiomes and core resistomes, respectively. **, *P* < 0.01; *, *P* < 0.05. (B) PCoA of microbiomes for subclustering. (C) PCoA of core resistomes for subclustering. Black arrows indicate further subclustering.

The regionalization of microbiomes and core resistomes in APM was further supported by in-depth analysis, which suggested that both APM microbiomes and core resistomes could be regionally subclustered at significant levels (Fig. 3). Intriguingly, both microbiomes and core resistomes shared the identical significant subclustering hierarchy. These findings led us to conclude that the regionalization of microbiomes and core resistomes followed the same pattern, further suggesting that the same factors drove the regionalization. This finding also indicated a correlation between microbiomes and core resistomes in APMs.

### Shaping environmental factors of APM microbiomes and core resistomes.

**FIG 4:**
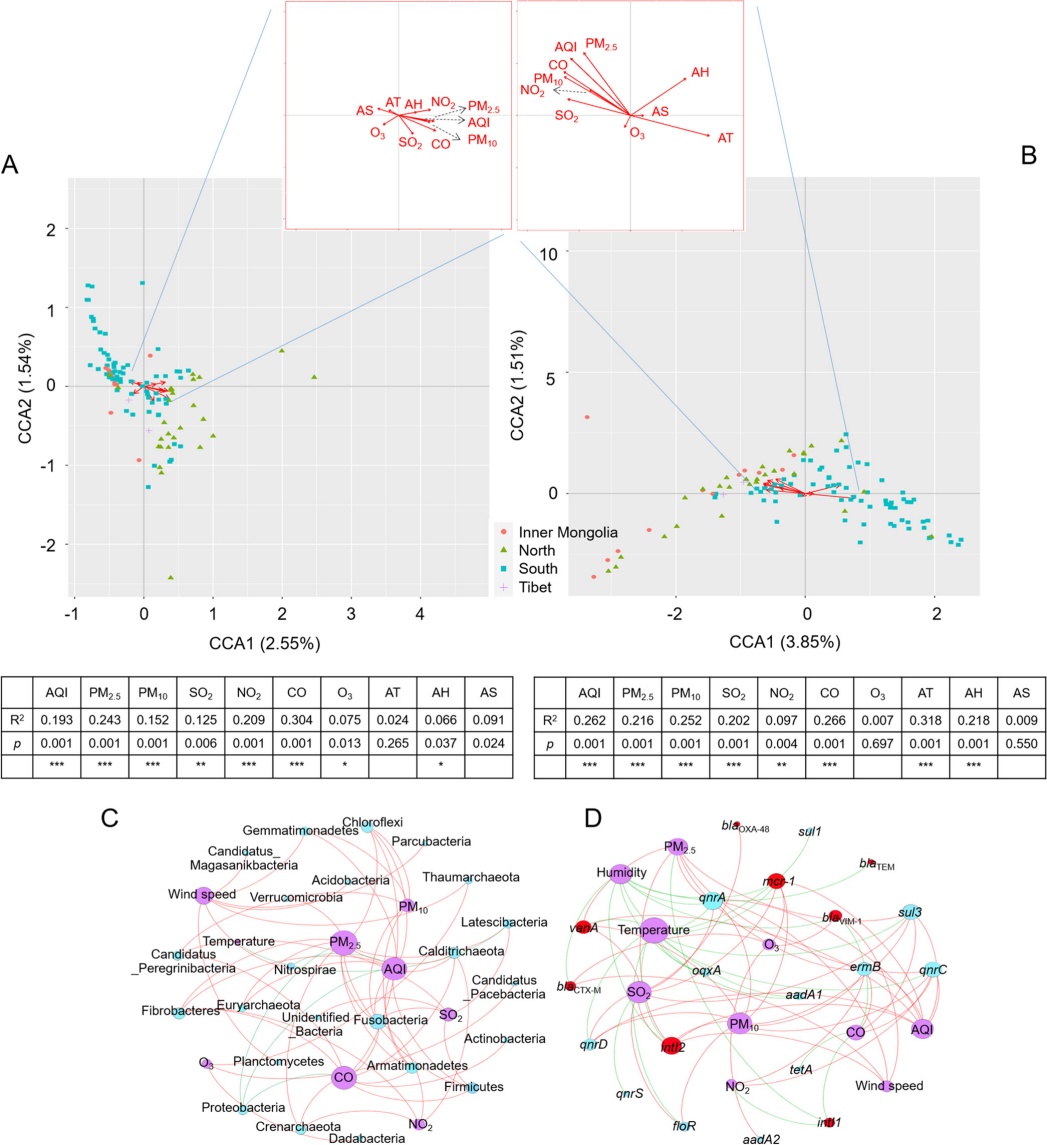
Impact of environmental parameters on APM microbiome and core resistomes compositions. (A) CCA of environmental parameters and microbial community structures, each point indicated an OUT. (B) CCA of environmental parameters and core resistome compositions, each point indicated one ARG. (C) Co-occurrence network analysis between microbial phyla and environmental parameters. (D) Co-occurrence network analysis between ARDs and environmental parameters. Purple circles indicate environmental parameters, red lines indicate a positive correlation, green lines indicate a negative correlation, and the sizes of circles indicate several correlation connections. AH, average humidity; AT, average temperature; AS, average wind speed; AQI, air quality index. ***, *P* < 0.001; **, *P* < 0.01; *, *P* < 0.05.

The impact of environmental parameters, including major pollutant levels on the compositions of APM microbiomes and core resistomes, was investigated with canonical correspondence analysis (CCA) and envfit analysis. The results showed that APM levels, including air quality index (AQI), PM_2.5_, and particulate matters with aerodynamic diameters smaller than 10 μm (PM_10_), together with most environmental pollutants (CO, SO_2_, NO_2_), significantly impacted APM microbiomes and core resistomes (Fig. 4A and B). For both microbiomes and core resistomes, the direction of impact of these environmental pollutants is similar. In general, although differences are present, the overall impact patterns of environmental parameters on APM microbiomes and core resistomes are consistent (Fig. 4A and B), confirming a correlation between them and corroborating with the shared regionalization subclustering hierarchy patterns found in this work.

Co-occurrence network analysis with Pearson correlation between environmental parameters and microbiome/resistome compositions was performed (Fig. 4C and D). Although bearing differences, environmental pollutants (CO, SO_2_, NO_2_) and APM levels (AQI, PM_2.5_, PM_10_) were dominant factors influencing the composition of the APM microbiomes and core resistomes. This agreed with the CCA and envfit analyses, where both APM microbiomes and core resistomes shared the same group of dominant influencing factors (AQI and environmental pollutants minus O_3_) whose influential strength could be estimated by the length of each vector representing each factor in CCA and R^2^ values in the envfit analyses. In addition, co-occurrence network analyses indicated that environmental pollutants and APM levels were positively correlated with bacterial phyla and ARDs. These results were consistent with the results found with the CCA in which these factors impacted APM microbiomes and core resistomes similarly. Meanwhile, humidity and temperature negatively correlated with the most influential ARGs in the co-occurrence network analysis. These findings also confirmed the results with the CCA and envfit analyses in which humidity and temperature were in a group of factors that oppositely impacted APM core resistomes to environmental pollutants and APM levels. The high consistency between CCA, envfit analysis, and co-occurrence network analysis suggested the robustness of the methods used in this work and the key suggestions that were made. Despite bearing several differences, APM microbiome and core resistome compositions were shaped by mostly the same dominant factors (environmental pollutants and APM levels), and they appeared to be correlated.

### Significant correlations between APM microbiome and core resistome compositions.

Using statistical methods comparing two data matrices, investigations on the correlations of APM-associated microbiomes and core resistomes were further attempted. To gain better comparability between the two data matrices representing microbiomes and core resistomes, the level of each microbial taxon (at the level of operational taxonomic units) and ARD was converted to relative abundance, whereas the total abundances of all taxa and ARDs were set to 1 (Fig. S1). The Procrustes analysis found a significant correlation between APM-associated microbiome and core resistome compositions (M^2^ = 0.963, *P = *0.031, permutations = 999, Fig. S2). This finding was further confirmed by the Mantel test comparing microbiomes and core resistomes where Bradyrhizobium elkanii was removed for its high abundance that strongly disturbs calculation (*R* = 0.06, *P = *0.008). In combination with the similar regionalization patterns of microbiomes and core resistomes in APMs, as well as their shared dominant driving environmental factors, these results clearly and strongly suggested that APM microbiomes and core resistomes were significantly correlated, an observation not reported previously to the best of our knowledge.

10.1128/msystems.00698-22.2 FIG S2 Procrustes analysis of the correlation between microbiomes and core resistomes. Download FIG S2, TIF file, 0.5 MB. Copyright © 2022 Li et al. 2022 Li et al. https://creativecommons.org/licenses/by/4.0/ This content is distributed under the terms of the Creative Commons Attribution 4.0 International license.

### Key players in the correlation between APM-associated microbiomes and core resistomes.

**FIG 5:**
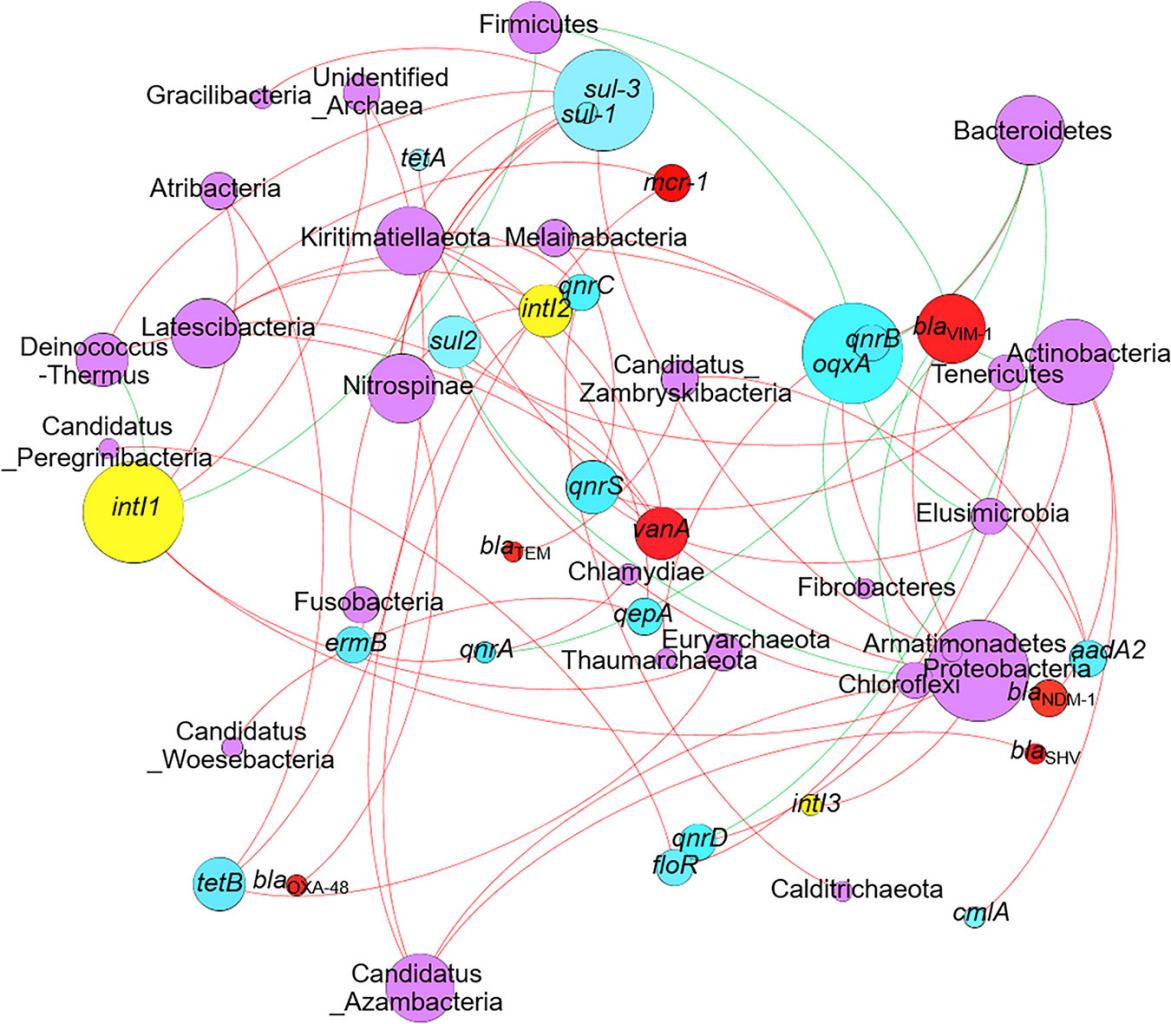
Co-occurrence network analysis of microbial phyla and ARDs. Purple circles indicate microbial phyla, yellow circles indicate integrase-encoding genes of integrons, blue circles indicate ARGs, red circles indicate the ARGs for last-line antibiotics, red lines indicate a positive correlation, green lines indicate negative correlation, and sizes of circles indicate the number of correlation connections.

ARDs were encoded by bacteria to carry out their functions. Therefore, microbiomes could be viewed as the “environment” for resistomes. With the establishment of significant correlations between APM-associated microbiomes and core resistomes, identifying key microbes that shape resistomes will help us to understand how airborne resistomes were formed. A co-occurrence network analysis with Pearson correlation between microbial phyla and ARDs was performed (Fig. 5). Unsurprisingly, the most common bacterial phyla, Proteobacteria, Actinobacteria, Bacteroides, and Firmicutes, played essential roles in this interaction network. These key phyla, however, appeared to play different roles in shaping resistomes because Firmicutes and Bacteroides were mostly negatively correlated with key ARDs. In contrast, Proteobacteria and Actinobacteria were positively correlated with key ARDs (Fig. 5). Several less common phyla, including Deinococcus-Thermus, Candidatus Azambacteria, Latescibacteria, Kiritimatiellaeota, and Nitrospinae, also played a role in resistome formation (Fig. 5). Intriguingly, they significantly correlated more often with ARDs targeting last-line antibiotics, including *mcr-1* (targeting polymyxin, correlated with Candidatus_Azambacteria, Latescibacteria) and *vanA* (targeting vancomycin, correlated with Deinococcus-Thermus, Latescibacteria, Kiritimatiellaeota). This phenomenon prompted us to wonder whether these less investigated bacterial groups played a more pronounced role in AMR targeting last-line antibiotics than originally expected.

From the perspective of ARDs, the Class 1 integron integrase-coding gene *intI1*, sulfonamide resistance gene *sul3*, and quinolone resistance gene *oqxA* played central roles in APM microbiome-resistome interactions (Fig. 5). This finding suggested the important roles of these three ARDs in shaping resistome and implied their wide prevalence in different bacterial phyla. Other common ARDs targeting quinolones, chloramphenicol, aminoglycosides, sulfonamides, tetracycline, and erythromycin also played a role. Surprisingly, although β-lactams were the most important antibiotics, ARGs targeting this class of antibiotics did not play a significant role in the interaction between APM-associated microbiomes and core resistomes. ARGs targeting last-line antibiotics, including *bla*_VIM-1_, *mcr-1*, and *vanA*, were found to be more important in resistomes than originally expected.

## DISCUSSION

The compositions of microbiomes are highly variable due to both the many environmental factors that influence microbes and the complex dynamic interactions between microbiomes. Many factors, including meteorological parameters (16), public transit (37), time (21), location (36), and human and animal activities (16, 36), were reported to impact APM microbiome composition. For instance, temperature, humidity, and environmental pollutants were significantly associated with the abundance of opportunistic pathogens in the APM microbiome, and increased human activity significantly altered the structure of the APM microbiomes (16). This high variability has long been troubling scientists as dictating a ‘normal’ microbiome and targeted regulation of microbiomes will be difficult. Results from this work showed that environmental parameters, including pollutant levels and APM levels, were the driving factors that shaped APM microbiomes. While the impact of pollutants is understandable because they can selectively influence bacterial growth, the impact of APM levels on microbiome compositions implied that the presence of APM could regulate microbiome compositions by primarily promoting the growth of bacteria (Fig. 4C), with the exception that PM_2.5_ was negatively correlated with Proteobacteria. Considering Proteobacteria is a primary bacterial phylum in environmental microbiomes, this negative correlation with APM levels may also imply that Proteobacteria is an essential target during the regulation of microbiomes by APMs. This regulation of microbiomes by APMs can be explained by the selective promotion of nutrient-favoring bacteria in high-APM environments as more nutrients are available in a high-APM atmosphere.

The clear and strong regionalization of APM microbiomes found in this work suggested that geological location is also a dominant driving factor in shaping microbiomes. He et al. (38) reported in 2018 that healthy human gut microbiomes show high levels of regional variation, suggesting that localized baselines for human gut microbiomes are necessary. In this work, with samples collected from 13 cities of four geologically close clusters in China, we found significant differences between microbiomes from different clusters (Northern cities, Southern cities, Tibet, Inner Mongolia) and significant differences between microbiomes of different cities in each cluster. Shenzhen was found to be clustered with Inner Mongolia and Tibet rather than other cities from Guangdong Province to which it belongs. We suspect this is because Shenzhen, like Inner Mongolia and Tibet, had a higher O_3_ level in the air than other Guangdong cities (*P* < 0.05; Table S1). This may contribute to the similar microbiomes and further similar resistomes in these three locations. The novel finding of the regionalization feature of APM microbiomes suggested that, like healthy human gut microbiomes, APM microbiomes were also highly regionally variable and could contribute to the regional variability of healthy human gut microbiomes. We suspected that other environmental microbiomes might also be highly regionally variable. The regionalization of healthy human gut microbiomes may be the result of the regionalization of environmental microbiomes.

The environment of APM microbiomes (pollutants, APM levels, geological locations) shapes the compositions of microbiomes. The APM microbiomes, on the other hand, can be viewed as the environments of resistomes. ARDs are primarily carried by bacteria. Meanwhile, the capability to carry ARDs differs significantly between bacterial species. For instance, Klebsiella pneumoniae, a widespread opportunistic pathogen, has been repeatedly reported to carry many ARDs (39). Therefore, theoretically, the APM microbiome compositions should shape or even decide APM resistome compositions. There are controversial reports on the correlation between environmental microbiomes and resistomes. Several reports found a significant correlation between microbiomes and resistomes in environments, such as wastewater treatment plants and seawater (40, 41). Meanwhile, a lack of correlation was reported between microbiomes and resistomes in the intestinal tracts of migration birds (42). Few reports have investigated the correlation between APM microbiomes and resistomes, except for a recent publication on Microbiome that collected emitted bioaerosols of a hospital (*n* = 19) in Guangzhou city and urban PM_2.5_ (*n* = 10) in the same city for analysis (43). A significant correlation was found between APM microbiomes and resistomes in the hospital but not for urban APM samples.

This work provided overwhelming evidence showing that APM microbiomes and resistomes were correlated. The same regionalization patterns were found for APM microbiomes and core resistomes. Shared driving environmental factors were found to shape APM microbiomes and core resistomes in the same patterns. Direct statistical methods (Procrustes analysis and Mantel tests) also showed a significant correlation between 112 APM microbiomes and core resistomes collected from 13 cities in four clusters that are geologically distant from one another. The closest sampling clusters (Shaanxi and Inner Mongolia) were over 700 km apart, while Lhasa was at least 1,700 km away from any other sampling location. We believe that the inconsistency of our findings and the recently published findings that microbiomes and resistomes in urban PM_2.5_ were not correlated in Guangzhou city was because of the relatively smaller number of samples taken in the published literature (*n* = 10) versus in this work (*n* = 112) and the lack of diversity in geological locations in the published literature (one city versus 13 cities in this work) (43).

Co-occurrence network analysis between microbiomes and resistomes revealed key taxa and key ARDs in their relationship (Fig. 5). The most common environmental bacterial phyla, including Proteobacteria, Bacteroides, Actinobacteria, Firmicutes, as well as common ARDs, including *intI1*, *oqxA*, and *sul-3* were found to be major factors shaping this relationship. Surprisingly, β-lactam resistance genes do not seem to be involved in the microbiome-resistome relationship. β-lactams are by far the most important and widely used antibiotics. The abundances of genes resistant to these antibiotics are high in almost every sample and every ecosystem. We believe the lack of correlation of β-lactam resistance genes with microbes was because of their high abundance. They are everywhere anyway. Therefore, the differences in the carriage of these ARGs by different microbes were already minor. Another surprising finding was the role of relatively uncommon phyla, such as Deinococcus-Thermus, Latescibacteria, Nitrospinae, Candidatus_Azambacteria, and the role of ARGs targeting last-line antibiotics, such as *mcr-1*, *vanA*, *bla*_NDM-1_, *bla*_OXA-48_, and more importantly their mutual interactions. These bacterial phyla do not make a large fraction of microbiomes, and ARGs targeting last-line antibiotics have been considered rare in the environment. Indeed, last-line antibiotics are considered the last medical option in treating antibiotic-resistant bacteria, and the presence of ARGs targeting them is often considered clinically catastrophic. Governments and medical facilities worldwide have made strict rules in using these antibiotics to prevent the emergence and spread of AMR against them. This finding, thus, revealed a previously neglected aspect that may be important in APM microbiomes and the shaping of resistomes. These relatively “rare” microbial groups may play an important role in carrying rare but clinically important ARGs. The microbial groups also play key roles in shaping resistomes and spreading resistance against last-line antibiotics, the last weapon in human’s arsenal against bacterial infection. These conclusions still need further verification by isolating microbes carrying these ARGs from APMs.

The transmission of infectious microbes via air is a common transmission pathway of microbial infection. Many infectious diseases, such as tuberculosis and severe acute respiratory syndrome (SARS), are known to be transmitted by short-distance airborne pathways. APM, an important detrimental air component, has been reported to be involved in the transmission of microbial infections in recent years. For instance, SARS coronavirus 2 (SARS-CoV-2) RNA was found on airborne particles in Bergamo, northern Italy (44), showing evidence that APMs can spread the virus causing the coronavirus disease 2019 (COVID-19). The potential for APMs to transmit pathogenic bacteria has also been demonstrated in published reports (16), supporting the theory that APM can promote bacterial pathogenesis. Besides pathogenic bacteria, AMR is also an essential part of bacterial pathogenesis. Antibiotic-resistant pathogen infections are more challenging to treat than antibiotic-sensitive pathogens, and ARGs have the potential to spread between cells, leading to an overall increase of antibiotic resistance in a given microbiome. This aspect of bacterial pathogenesis has also been investigated in APMs (16, 21, 36), confirming the high prevalence of APM-associated ARDs. Therefore, both the compositions of microbiomes and resistomes contribute to and impact bacterial pathogenesis resulting from the proliferation of APM. These are important aspects to consider for the suppression of this important bacterial pathogenesis transmission pathway.

**FIG 6:**
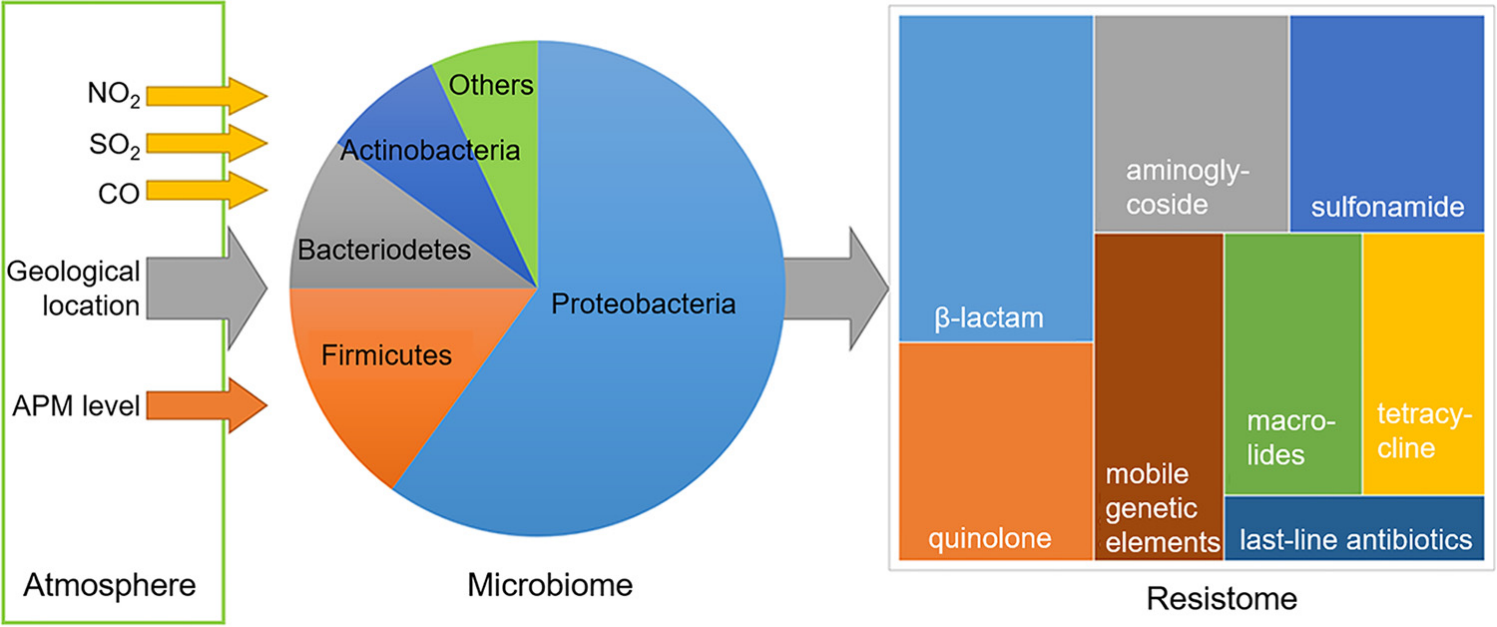
Model of dynamics in shaping APM microbiomes and resistomes. The direction of arrows indicates the impact of shaping factors on microbiome or resistomes compositions.

Results obtained in this work lead to the proposal of a model in the dynamics of APM-associated bacterial pathogenesis (Fig. 6). Environmental pollutants, including NO_2_, SO_2_, and CO, APM levels, and geological locations are key factors in shaping the microbiomes associated with APM. The dynamics of APM microbiomes further shaped the composition of APM resistomes, in which both common and rare bacterial taxa made contributions that may not be proportional to the abundance of each taxon. It needs to be addressed that, despite strong evidence supporting the primary findings of this work, we believe this work only reports a small fraction of environmental factors that shape APM microbiomes and resistomes, including a large collection of chemicals in APMs, the dynamics of airflow, and biological behaviors that lead to the input of human, animal, or even plant-originated microbes. In addition, the omics-based approach carried out in this work cannot replace wet-lab investigations of bacterium-bacterium and bacterium-environment interactions in APMs. Therefore, we believe further, more comprehensive, multicenter, long-term surveillance is needed to elucidate how APM microbiomes and resistomes are determined.

In conclusion, with APM samples collected from 13 cities located in geologically distant clusters in China, we found strong regionalization for both APM microbiomes and core resistomes. A similar set of environmental parameters, including environment pollutant levels and APM levels, were the primary driving factors that shaped both APM microbiomes and core resistomes. These findings, together with direct statistical analysis showing significant correlations between APM microbiomes and core resistomes, provided overwhelming evidence suggesting microbiomes and core resistomes were correlated in APM. Key taxa and ARDs in the interaction of microbiomes and resistomes were found, suggesting the role of abundant and relatively rare taxa and the role of ARDs in common and last-line antibiotics. These results support the theory that environmental parameters drive the formation of APM microbiomes, and APM microbiomes further drive the formation of APM resistomes.

## MATERIALS AND METHODS

### APM sampling.

A total of 112 urban APM samples were collected from 13 cities in China, including five cities in Shaanxi Province in Northern China, six cities in Guangdong Province and neighboring Hong Kong Special Administration Region in Southern China, as well as two outlying cities in Tibetan Autonomous Region and Inner Mongolia Autonomous Region of China between January 2017 and March 2018 (Fig. 1A and Table S1). The environmental parameters of the samples were collected from https://www.aqistudy.cn/. APM samples were collected using medium volume air samplers equipped with quartz fiber filters and set at PM_2.5_ and PM_10_ modes for the sampling. Samples were collected every 12 h at an airflow rate of 100 L/min.

### 16S rRNA gene amplicon sequencing.

APM-containing quartz fiber filters were cut into four equal pieces. One of the pieces was buffered with 200 mM sterile phosphate buffer (pH = 7.4), followed by DNA extraction (16) using a Plant Genomic DNA kit (Tiangen Biotech Co., Ltd., Beijing, China). Total DNA was extracted from APM samples. Further, the DNA was subjected to high-throughput sequencing where the V4 to V5 regions of 16S rRNA genes were sequenced with an Illumina HiSeq 2500 sequencer (Illumina, Inc., San Diego, CA, US). Effective tags from raw sequence data were obtained using FLASh v1.2.7 and QIIME v1.7.0 software (45, 46). Chimeric sequences were removed using the UCHIME algorithm (47). Uparse v7.0.1001 was used to cluster sequences into operational taxonomy units with an identity cutoff of 97% (48).

### Quantitative real-time PCR.

APM core resistomes, including three mobile genetic elements and 29 antibiotic resistance genes (ARGs), were determined with qRT-PCR (Table S2 andS3). qRT-PCR standard curves for the targeted genes were constructed. Primers used for qPCRs are shown in Table S2. Except for the *catB3* and *bla*_CTX-M_ that used qCatB3-F/qCatB3-R and qCTXM-F/qCTXM-R primers, all other PCR primers and positive controls used in this study were the same as our previous study (16). Targeted amplicons were purified by a Universal DNA purification kit (Tiangen Biotech Co., Ltd., Beijing, China), cloned into a pMD 19-T vector (TaKaRa, Dalian, China), and transferred into Escherichia coli DH5α competent cells. Plasmids containing targeted genes were selected by blue-white screening and extracted by the TIANprep Mini Plasmid kit (Tiangen Biotech Co., Ltd., Beijing, China). The sequences were verified by Sanger Sequencing in Beijing Genomics Institute (BGI, China). qPCR assays were performed using SYBR green-based approach on a StepOnePlus real-time PCR system (Applied Biosystems, USA) for the determination of the relative content of ARGs following previously published protocols (36, 49).

### Bioinformatics analysis and statistics.

The compositions of microbiomes and core resistomes were normalized so that all bacterial groups and ARGs add up to one in each data set for further analysis. The correlation analysis was performed to analyze the relationship between bacterial community composition, environmental parameters, and AMR-determinants via two-tailed Pearson correlation and Gephi software (version 0.9.2). The PCoA (principal coordinates analysis) and ANOSIM (analysis of similarities) methods were performed to find out whether the microbiomes (unweighted Unifrac distance) and core resistomes (Bray-Curtis distance) groups are significantly different. Moreover, the correlation analysis to analyze the relationship between microbial community composition and core resistomes was performed via Procrustes analysis and Mantel tests using the Vegan package on the R platform. Canonical correspondence analysis (CCA) and envfit analyses were performed with the Vegan package on the R platform. LefSe (LDA effect size) analysis was performed at http://huttenhower.sph.harvard.edu/galaxy/.

### Data availability.

The 16S rRNA gene amplicon sequencing data can be found in the National Omics Data Encyclopedia (NODE) database under Project ID OEP003304. All other data are included in this article or supplemental material.
