# Supplementary figures and images for: Regionalization and Shaping Factors for Microbiomes and Core Resistomes in Atmospheric Particulate Matters
Source: mSystems. 2022 Sep 26;7(5):e00698-22. doi: 10.1128/msystems.00698-22 (PMC9600985; doi:10.1128/msystems.00698-22)

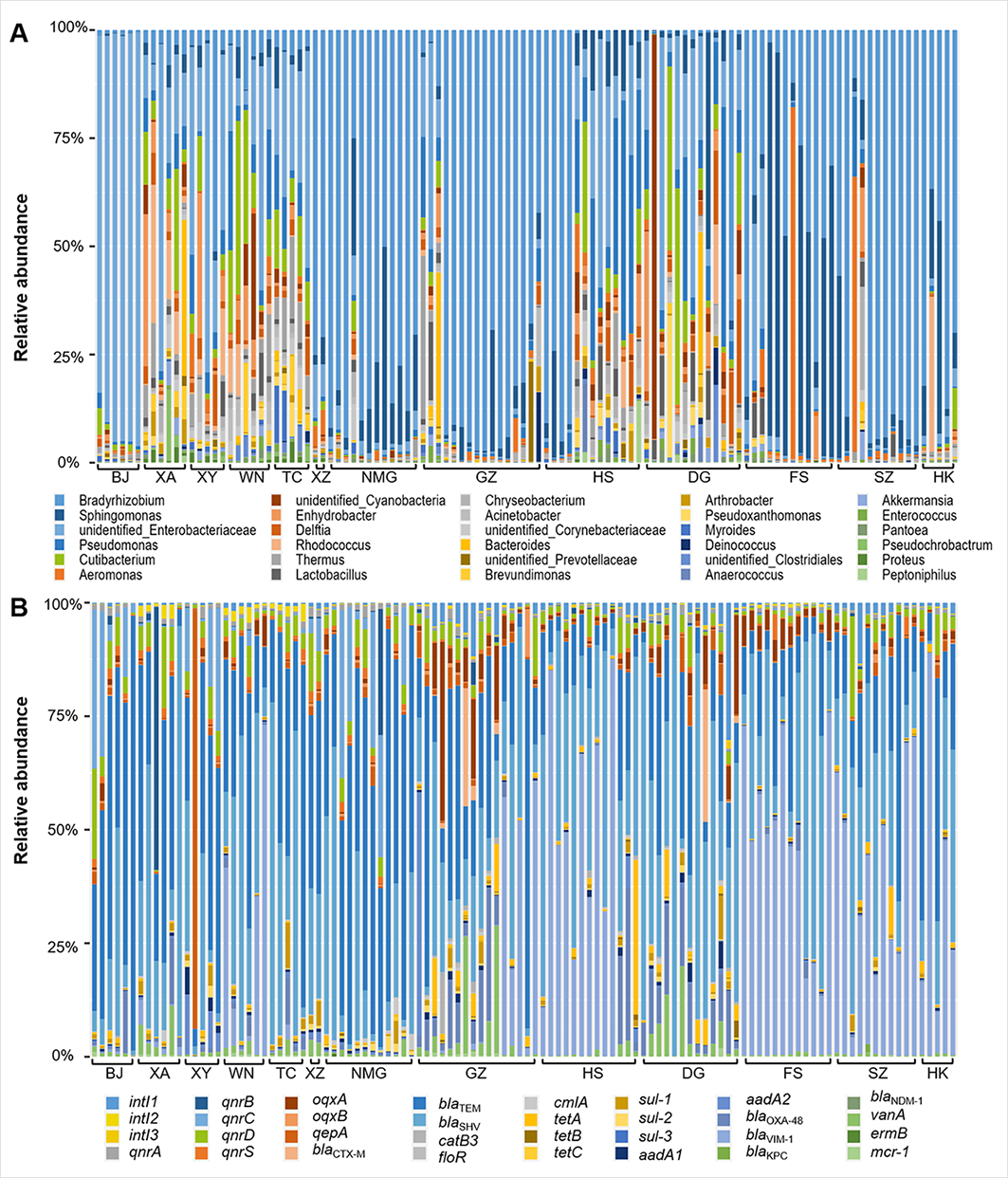

Supplement: FIG S1 [file msystems.00698-22-s0001.tif]

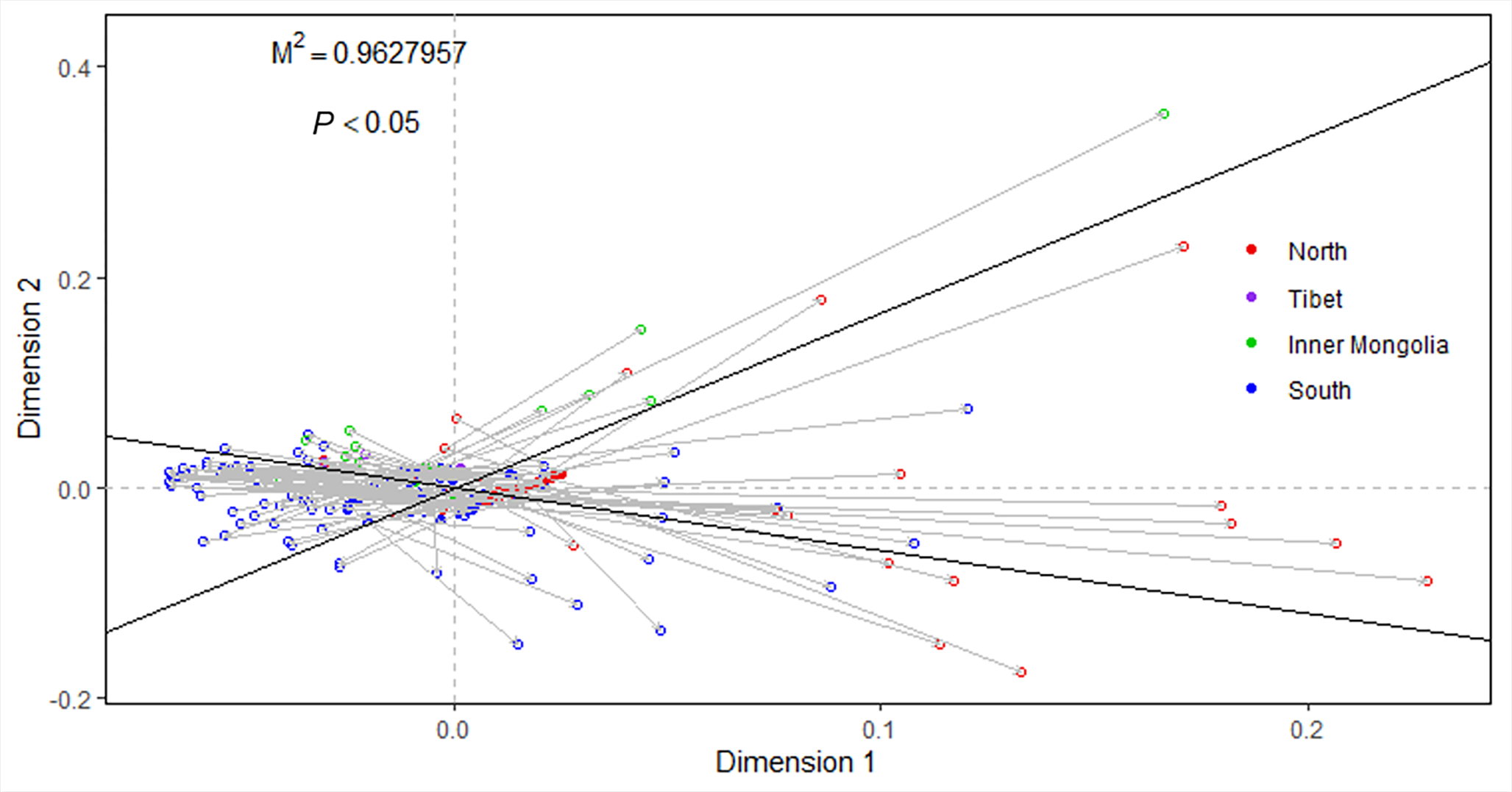

Supplement: FIG S2 [file msystems.00698-22-s0002.tif]
