# Supplementary material for: Regionalization and Shaping Factors for Microbiomes and Core Resistomes in Atmospheric Particulate Matters
Source: mSystems. 2022 Sep 26;7(5):e00698-22. doi: 10.1128/msystems.00698-22 (PMC9600985; doi:10.1128/msystems.00698-22)
Supplement: TABLE S2 [file msystems.00698-22-s0004.docx]

| **Class** | **Gene** | **Primer** | **Primer sequence (5′-3′)** | **Size (bp)** | **Reference** |
| --- | --- | --- | --- | --- | --- |
| Integrase-coding genes | *intI1* | qIntI1-F | GCCTTGATGTTACCCGAGAG | 196 | 1 |
|  |  | qIntI1-R | GATCGGTCGAATGCGTGT |  |  |
|  | *int2* | qInt2-F | GACGGCTACCCTCTGTTATCTC | 195 | 1 |
|  |  | qInt2-R | TGCTTTTCCCACCCTTACC |  |  |
|  | *int3* | qInt3-F | CAGACGTTGCTTTCGCACAT | 112 | This study |
|  |  | qInt3-R | AATCCACATCCTTGACCCGC |  |  |
| Quinolone resistant genes | *qnrA* | qQnrA-F | ATTTCTCACGCCAGGATTTG | 158 | 2 |
|  |  | qQnrA-R | CAGATCGGCATAGCTGAAG |  |  |
|  | *qnrB* | qQnrB-F | GGMATHGAAATTCGCCACTG | 245 | 2 |
|  |  | qQnrB-R | TTYGCBGYYCGCCAGTCGAA |  |  |
|  | *qnrC* | qQnrC-F | CAATGGCGAATTTCCAAG | 139 | 3 |
|  |  | qQnrC-R | ACCCGTAATGTAAGCAGAGC |  |  |
|  | *qnrD* | qQnrD-F | GAGCTGATTTTCGAGGGGCTA | 190 | 3 |
|  |  | qQnrD-R | AGATCGGAGCCACGAAACAC |  |  |
|  | *qnrS* | qQnrS-F | GACGTGCTAACTTGCGTGAT | 118 | 2 |
|  |  | qQnrS-R | TGGCATTGTTGGAAACTTG |  |  |
|  | *oqxA* | qOqxA-F | GGGATAGTTTTAACGGTCGCATTG | 266 | 4 |
|  |  | qOqxA-R | TTCACGGGAGACGAGGTTGGT |  |  |
|  | *oqxB* | qOqxB-F | TCCTGATCTCCATTAACGCCCA | 130 | 5 |
|  |  | qOqxB-R | ACCGGAACCCATCTCGATGC |  |  |
|  | *qepA* | qQepA-F | TGTGGATCGCCGCGTTTT | 124 | 3 |
|  |  | qQepA-R | GCCAGCGTCAGCAGCATCA |  |  |
| β-lactam resistant genes | *bla*_CTX-M_ | qCTXM-F | ATTCCRGGCGAYCCGCGTGATACC | 205 | 6 |
|  |  | qCTXM-R | ACCGCGATATCGTTGGTGGTGCCAT |  |  |
|  | *bla*_TEM_ | qTEM-F | GCKGCCAACTTACTTCTGACAACG | 247 | 7 |
|  |  | qTEM-R | CTTTATCCGCCTCCATCCAGTCTA |  |  |
|  | *bla*_SHV_ | qSHV-F | GATGAACGCTTTCCCATGATG | 213 | 8 |
|  |  | qSHV-R | CGCTGTTATCGCTCATGGTAA |  |  |
| Chloramphenicol resistant genes | *catB3* | qCatB3-F | TGGGGCTTCCTTTATCGTGG | 188 | This study |
|  |  | qCatB3-R | TGATTCCGGGCATGACCATT |  |  |
|  | *floR* | qFloR-F | GAGGGTGTCGTCATCTACGG | 138 | 9 |
|  |  | qFloR-R | GAGCATCGCCAGTATAGCCA |  |  |
|  | *cmlA* | qCmlA-F | GCTGCTACTCCCCGTTAAGT | 117 | 9 |
|  |  | qCmlA-R | TGCCTGCCCATCATTAGTCC |  |  |
| Sulfonamide resistant genes | *sul-1* | qSul-1-F | CGCACCGGAAACATCGCTGCAC | 162 | 10 |
|  |  | qSul-1-R | TGAAGTTCCGCCGCAAGGCTCG |  |  |
|  | *sul-2* | qSul-2-F | TCATCTGCCAAACTCGTCGTTA | 105 | 10 |
|  |  | qSul-2-R | GTCAAAGAACGCCGCAATGT |  |  |
|  | *sul-3* | qSul-3-F | TTCGTTCACGCTTTACACCAGC | 127 | 10 |
|  |  | qSul-3-R | TCCGTTCAGCGAATTGGTGCAG |  |  |
| Tetracycline resistant genes | *tetA* | qTetA-F | CGCTGCAAGCAATGTTGTCC | 121 | This study |
|  |  | qTetA-R | TAGATCGCCGTGAAGAGGAGG |  |  |
|  | *tetB* | qTetB-F | GAAGTAGGGGTTGAGACGCA | 111 | This study |
|  |  | qTetB-R | TGCGGGAATTTGGCCTATCA |  |  |
|  | *tetC* | qTetC-F | GGCGAGAAGCAGGCCATTAT | 118 | This study |
|  |  | qTetC-R | CGCCGGAAGCGAGAAGAATC |  |  |
| Polymyxin resistant gene | *mcr-1* | qMcr1-F | GGGCCTGCGTATTTTAAGCG | 183 | 11 |
|  |  | qMcr1-R | CATAGGCATTGCTGTGCGTC |  |  |
| Aminoglycoside resistant genes | *aadA1* | qAadA1-F | AGCTAAGCGCGAACTGCAAT | 67 | 12 |
|  |  | qAadA1-R | TGGCTCGAAGATACCTGCAA |  |  |
|  | *aadA2* | qAadA2-F | CTTGTCGTGCATGACGACATC | 101 | 13 |
|  |  | qAadA2-R | TCGAAGATACCCGCAAGAATG |  |  |
| Erythromycin resistant gene | *ermB* | qErmB-F | GATACCGTTTACGAAATTGG | 364 | 9 |
|  |  | qErmB-R | GAATCGAGACTTGAGTGTGC |  |  |
| Carbapenem resistant genes | *bla*_KPC_ | qKPC-F | TTACGGCAAAAATGCGCTGG | 201 | This study |
|  |  | qKPC-R | TCCAGACGGAACGTGGTATC |  |  |
|  | *bla*_NDM-1_ | qNDM1-F | GCCCAGATCCTCAACTGGAT | 135 | This study |
|  |  | qNDM1-R | CGCATTGGCATAAGTCGCAA |  |  |
|  | *bla*_VIM-1_ | qVIM1-F | GGTCCAGAACCTTGACCGAA | 206 | This study |
|  |  | qVIM1-R | GTGGGTGCGGCATAACTTTG |  |  |
|  | *bla*_OXA-48_ | q OXA48-F | CGGTAGCAAAGGAATGGCAA | 133 | 14 |
|  |  | qOXA48-R | TGGTTCGCCCGTTTAAGATT |  |  |
| Vancomycin resistant gene | *vanA* | qVanA-F | AAAAGGCTCTGAAAACGCAGTTAT | 150 | 12 |
|  |  | qVanA-R | CGGCCGTTATCTTGTAAAAACAT |  |  |
| 16S rDNA | *16SrRNA* | q16S-F | CCCAGATGGGATTAGCTTGT | 106 | 16 |
|  |  | q16S-R | TCTGGACCGTGTCTCAGTTC |  |  |

**References**

1. Barraud O, Baclet MC, Denis F, Ploy MC. 2010. Quantitative multiplex real-time PCR for detecting class 1, 2 and 3 integrons. J Antimicrob Chemother 65(8):1642-1645.

2. Marti, E, Balcázar JL. 2013. Real-Time PCR assays for quantification of *qnr* genes in environmental water samples and chicken feces. Appl Environ Microbiol 79(5):1743-1745.

3. Yan L, Liu D, Wang XH, Wang Y, Zhang B, Wang M, et al. 2017. Bacterial plasmid-mediated quinolone resistance genes in aquatic environments in China. Sci Rep 7:40610.

4. Guo W, Hao H, Dai M, Wang Y, Huang L, Peng D, et al. 2012. Development of quinoxaline 1, 4-dioxides resistance in *Escherichia coli* and molecular change under resistance selection. Plos One 7(8):e43322.

5. Li J, Wang T, Shao B, Shen J, Wang S, Wu Y. 2012. Plasmid-mediated quinolone resistance genes and antibiotic residues in wastewater and soil adjacent to swine feedlots: potential transfer to agricultural lands. Environ Health Perspect 120(8):1144-1149.

6. Fujita S, Yosizaki K, Ogushi T, Uechi K, Takemori Y, Senda Y. 2011. Rapid identification of gram-negative bacteria with and without CTX-M extended-spectrum β-lactamase from positive blood culture bottles by PCR followed by microchip gel electrophoresis. J Clin Microbiol 49(4):1483-1488.

7. Xi C, Zhang Y, Marrs CF, Ye W, Simon C, Foxman B, et al. 2009. Prevalence of antibiotic resistance in drinking water treatment and distribution systems. Appl Environ Microbiol 75(17): 5714-5718.

8. Kim JS, Kim J, Kim SJ, Jeon SE, Oh KH, Cho SH, et al. 2014. Characterization of CTX-M-type extended-spectrum beta-lactamase-producing diarrheagenic *Escherichia coli* isolates in the Republic of Korea during 2008-2011. J Microbiol Biotechnol 24(3): 421-426.

9. Wang M, Shen W, Yan L, Wang XH, Xu H. 2017. Stepwise impact of urban wastewater treatment on the bacterial community structure, antibiotic contents, and prevalence of antimicrobial resistance. Environ Pollut 231:1578-1585.

10. Jiang L, Hu X, Xu T, Zhang H, Sheng D, Yin D. 2013. Prevalence of antibiotic resistance genes and their relationship with antibiotics in the Huangpu River and the drinking water sources, Shanghai, China. Sci Total Environ 458-460: 267-272.

11. Hembach N, Schmid F, Alexander J, Hiller C, Rogall ET, Schwartz T. 2017. Occurrence of the *mcr-1* colistin resistance gene and other clinically relevant antibiotic resistance genes in microbial populations at different municipal wastewater treatment plants in Germany. Front Microbiol 8:1282.

12. Su JQ, Wei B, Ou-Yang WY, Huang FY, Zhao Y, Xu HJ, et al. 2015. Antibiotic resistome and its association with bacterial communities during sewage sludge composting. Environ Sci Technol 49(12): 7356-7363.

13. Muziasari WI, Pitkänen LK, Sørum H, Stedtfeld RD, Tiedje JM, Virta M. 2017. The resistome of farmed fish feces contributes to the enrichment of antibiotic resistance genes in sediments below Baltic Sea fish farms. Front Microbiol 7:2137.

14. Brown-Jaque M, Calero-Cáceres W, Espinal P, Rodríguez-Navarro J, Miró E, González-López JJ, et al. 2018. Antibiotic resistance genes in phage particles isolated from human faeces and induced from clinical bacterial isolates. Int J Antimicrob Agents 51(3):434-442.

15. Poirel L, Walsh TR, Cuvillier V, Nordmann P. 2011. Multiplex PCR for detection of acquired carbapenemase genes. Diagn Microbiol Infect Dis 70(1):119-123.

16. Kim JY, Lee JL. 2014. Multipurpose assessment for the quantification of *Vibrio spp.* and total bacteria in fish and seawater using multiplex real-time polymerase chain reaction. J Sci Food Agric 94(13): 2807-2817.
