# Supplementary material for: Regionalization and Shaping Factors for Microbiomes and Core Resistomes in Atmospheric Particulate Matters
Source: mSystems. 2022 Sep 26;7(5):e00698-22. doi: 10.1128/msystems.00698-22 (PMC9600985; doi:10.1128/msystems.00698-22)
Supplement: TABLE S3 [file msystems.00698-22-s0005.docx]

| **Gene** | **Standard curve** | **R^2^** | **Amplication efficiency** |
| --- | --- | --- | --- |
| *16SrRNA* | y = 2E+15e^-0.713x^ | 0.9978 | 1.04 |
| *intI1* | y = 1E+13e^-0.726x^ | 0.9998 | 1.07 |
| *intI2* | y = 1E+11e^-0.658x^ | 0.9989 | 0.93 |
| *intI3* | y = 7E+10e^-0.729x^ | 0.9902 | 1.09 |
| *qnrA* | y = 2E+11e^-0.642x^ | 0.9974 | 0.90 |
| *qnrB* | y = 3E+12e^-0.740x^ | 0.9974 | 1.10 |
| *qnrC* | y = 3E+11e^-0.713x^ | 0.9999 | 1.04 |
| *qnrD* | y = 7E+11e^-0.642x^ | 0.9942 | 0.91 |
| *qnrS* | y = 4E+11e^-0.652x^ | 0.9993 | 0.92 |
| *oqxA* | y = 9E+10e^-0.707x^ | 0.9912 | 1.04 |
| *oqxB* | y = 2E+11e^-0.669x^ | 0.9979 | 0.96 |
| *qepA* | y = 5E+11e^-0.642x^ | 0.9986 | 0.90 |
| *bla*_CTX-M_ | y = 4E+10e^-0.732x^ | 0.9982 | 1.08 |
| *bla*_SHV_ | y = 5E+13e^-0.641x^ | 0.9985 | 0.90 |
| *bla*_TEM_ | y = 6E+12e^-0.645x^ | 0.9995 | 0.91 |
| *catB3* | y = 1E+11e^-0.736x^ | 0.9977 | 1.09 |
| *floR* | y = 5E+10e^-0.700x^ | 0.9996 | 1.02 |
| *cmlA* | y = 5E+10e^-0.640x^ | 0.9996 | 0.90 |
| *sul-1* | y = 7E+10e^-0.638x^ | 0.9999 | 0.90 |
| *sul-2* | y = 9E+10e^-0.692x^ | 0.9995 | 1.00 |
| *sul-3* | y = 4E+10e^-0.673x^ | 0.9976 | 0.96 |
| *aadA1* | y = 3E+11e^-0.676x^ | 0.9982 | 0.97 |
| *aadA2* | y = 8E+10e^-0.642x^ | 0.9993 | 0.90 |
| *ermB* | y = 2E+11e^-0.654x^ | 0.9956 | 0.93 |
| *tetA* | y = 9E+10e^-0.449x^ | 0.9931 | 0.90 |
| *tetB* | y = 6E+10e^-0.669x^ | 0.9923 | 0.96 |
| *tetC* | y = 2E+11e^-0.642x^ | 0.9983 | 0.90 |
| *mcr-1* | y = 3E+11e^-0.647x^ | 0.9991 | 0.91 |
| *bla*_KPC_ | y = 4E+10e^-0.671x^ | 0.9970 | 0.96 |
| *bla*_NDM1_ | y = 3E+10e^-0.665x^ | 0.9900 | 0.96 |
| *bla*_VIM-1_ | y = 1E+12e^-0.707x^ | 0.9982 | 1.03 |
| *bla*_OXA-48_ | y = 1E+11e^-0.669x^ | 0.9996 | 0.95 |
| *vanA* | y = 5E+11e^-0.647x^ | 0.9989 | 0.91 |
